# Behavioral, computational, and neuroimaging studies of acquired apraxia of speech

**DOI:** 10.3389/fnhum.2014.00892

**Published:** 2014-11-03

**Authors:** Kirrie J. Ballard, Jason A. Tourville, Donald A. Robin

**Affiliations:** ^1^Speech Pathology, University of SydneyLidcombe, NSW, Australia; ^2^Department of Speech, Language, and Hearing Sciences, Boston UniversityBoston, MA, USA; ^3^Center for Computational Neuroscience and Neural Technology (CompNet), Boston UniversityBoston, MA, USA; ^4^Departments of Neurology, Radiology, Biomedical Engineering, Research Imaging Institute, University of Texas Health Science Center San AntonioSan Antonio, TX, USA; ^5^Biomedical Engineering, Honor’s College, University of Texas at San AntonioTX, USA

**Keywords:** apraxia of speech, speech motor control, DIVA, feedforward control, feedback control, inferior frontal gyrus (IFG), premotor cortex

## Abstract

A critical examination of speech motor control depends on an in-depth understanding of network connectivity associated with Brodmann areas 44 and 45 and surrounding cortices. Damage to these areas has been associated with two conditions—the speech motor programming disorder apraxia of speech (AOS) and the linguistic/grammatical disorder of Broca’s aphasia. Here we focus on AOS, which is most commonly associated with damage to posterior Broca’s area (BA) and adjacent cortex. We provide an overview of our own studies into the nature of AOS, including behavioral and neuroimaging methods, to explore components of the speech motor network that are associated with normal and disordered speech motor programming in AOS. Behavioral, neuroimaging, and computational modeling studies are indicating that AOS is associated with impairment in learning feedforward models and/or implementing feedback mechanisms and with the functional contribution of BA6. While functional connectivity methods are not yet routinely applied to the study of AOS, we highlight the need for focusing on the functional impact of localized lesions throughout the speech network, as well as larger scale comparative studies to distinguish the unique behavioral and neurological signature of AOS. By coupling these methods with neural network models, we have a powerful set of tools to improve our understanding of the neural mechanisms that underlie AOS, and speech production generally.

## Introduction

Apraxia of speech (AOS) has been described as a disorder affecting the ability to translate well-formed and filled phonological frames into accurate movement programs to generate speech (McNeil et al., 2009). The resulting intra- and inter-articulator timing and spatial errors give rise to the core perceptual features used for clinical diagnosis: increased segment and inter—segment durations, distorted phonemes, consistent error type across repeated productions of words, segmentation of syllables, and more equal stress over words and/or sentences. Given that over 90% of individuals with stroke-related AOS have some degree of Broca’s aphasia (Duffy, 2005), the disorder has long been associated with Broca’s area (BA44/45). However, a range of candidate sites have been proposed including lateral premotor cortex (BA6), anterior insula, supplementary motor area, somatosensory cortex, supramarginal gyrus, and basal ganglia implicating a distributed neural network underpinning speech production (e.g., see Duffy, 2005; Robin et al., 2008b; Ziegler, 2008; Ackermann and Ziegler, 2010, for reviews).

To provide a systematic framework for investigating the nature of the impairment in AOS, researchers have long employed published theories and models of limb and speech motor control. These have included the Schema Theory of Speech Motor Control and Learning (e.g., Schmidt and Lee, 1999), Dynamic Systems Theory (Kelso, 1995), Bayesian Decision Theory (e.g., Körding and Wolpert, 2006; Franklin and Wolpert, 2011), Gestural Phonology (Browman and Goldstein, 1989), and the Directions into Velocities of Articulators (DIVA) model of speech motor control (Guenther, 2006; Guenther et al., 2006). Many of these theories and models share a basic set of feedforward and feedback control mechanisms that include forward models of movement, efference copy, sensory feedback, and sensorimotor integration. Briefly, a movement goal is identified (e.g., grasping a cup, or producing a syllable of speech), a motor program or command for that movement is activated and initiated; simultaneously an efference copy of that command is generated and used to construct a forward model, or prediction of the expected sensory feedback from the issued motor command (Wolpert and Kawato, 1998). As the movement unfolds, the actual sensory consequences are compared to the forward model; if a mismatch is detected, error signals arise and, through sensorimotor integration, corrective motor commands are generated that modify the ongoing movement, compensating for prediction errors or unexpected external perturbations. The error signals are also used to update the “stored” feedforward motor commands, thereby improving the accuracy of future attempts to perform that motor task; that is, feedback error signals serve as a basis for motor learning or adaptation.

These basic mechanisms have been mapped onto anatomical sites and neural pathways through both animal and human structural and functional imaging studies, and further explored in computational studies of motor learning and simulated lesioning of networks. Studies of human stroke, including individuals with AOS, have provided both raw data for building these theories as well as robust tests of their biological plausibility and explanatory power. Consistent with the hypotheses of several candidate neuroanatomical lesion sites, questions regarding the nature of AOS have covered almost all stages of motor control. Researchers have asked whether the neurological damage in this disorder is affecting the integrity of existing stored speech motor programs, forward modeling, the feedback processes of error detection and/or correction, gradual tuning of motor programs, and/or producing sequences of motor programs.

Here, we review and integrate several lines of research that address the nature of the impairment in AOS; from perspectives of surface behavior, neuroimaging, and computational modeling. Studies of neural connectivity in AOS are only just emerging but one can hypothesize that alterations in damaged area(s) result in changes in network coupling properties, based on anatomical and functional modifications and adaptive processes that re-organize the sensory-motor neural system.

## Behavioral studies of AOS: disorder of feedback or feedforward control?

Behavioral paradigms designed specifically to test hypotheses about the nature of AOS and the role of feedback and feedforward motor control processes have included jaw visuomotor tracking, auditory perturbations during speech, and reaction time tasks.

### Visuomotor tracking

One of the first studies attempting specifically to test the integrity of feedback and feedforward motor control systems in AOS was that of Hageman et al. (1994). They used a visuo-motor tracking paradigm, whereby individuals use their jaw or lip to track sinusoidal movements of a target cursor on a computer screen. For each trial, the target moved at one of three frequencies (0.3, 0.6, or 0.9 Hz) or in an unpredictable fashion and was calibrated to 10 mm maximum jaw excursion per cycle. It has been argued that healthy adults rapidly develop an accurate motor program for the periodic jaw or lip open-close movement, allowing them to perform smooth movements with only occasional checking against the target for accuracy. Several studies now have reported that individuals with AOS, with or without orofacial apraxia, perform more poorly than healthy age-matched control participants and individuals with aphasia in tracking sinusoidal targets, which are moving in a highly predictable fashion (Hageman et al., 1994; Clark and Robin, 1998; Ballard and Robin, 2007; Robin et al., 2008a). Specifically, individuals with AOS are less accurate and more variable in matching the target frequency and amplitude of open-close movements, compared to age-matched healthy adults. Notably, individuals with AOS in those early studies were reported to perform similarly to healthy control participants when tracking a cursor that was moving in an *unpredictable* fashion. It was argued that individuals with AOS have difficulty building forward models of movement and so, in tracking sinusoidal targets, they were forced to rely on feedback control and so movements were less accurate and smooth than healthy adults. However, AOS and healthy speakers performed similarly on tracking unpredictable targets where both groups are forced to operate under feedback control as there is no pattern to learn. This implied that feedback-based motor control for this task was intact in the people with AOS. Of interest, Robin et al. (2008a) found that visuo-motor tracking performance for predictable signals by AOS speakers correlated well with measures of their speech accuracy, lending support to the hypothesis that AOS involves impairment in learning forward models (i.e., sensory predictions) and implementing feedforward control.

To further test the hypothesis of impaired forward modeling, Ballard and Robin (2007) used a variation on visuomotor tracking, called “pseudo-tracking” and compared it to the standard tracking tasks described above. Pseudo-tracking involves displaying the target cursor for a brief training period, then removing it and having the participant continue moving the jaw in the same pattern. Performance without the target signal visible revealed the frequency and amplitude accuracy of the movement program that the person developed during the training period. Two important differences were noted for pseudo-tracking compared with regular tracking in individuals with AOS: (a) the frequency of their jaw movement was less variable in pseudo-tracking, when the target signal was not visible; and (b) while there was evidence that they did develop a motor program of the sinusoidal movement, the specification of amplitude and frequency parameters was inaccurate relative to controls.

Based on these studies, Ballard and Robin (2007) proposed two explanations of apraxic speakers’ performance. First, these individuals may rely more heavily on feedback due to difficulty in developing the motor program to match the movement of the cursor. This suggests either impaired integration of sensory feedback during the training period for modifying, or learning, the required movement pattern, or poor retention of modifications. Second, they may be less able to inhibit attention to the feedback, constantly comparing their jaw movement to the target cursor and making adjustments, with feedback disrupting smooth programmed movement. It is also possible that error detection processes are inaccurate and/or slowed which would further disrupt ongoing movement, however, this is less likely given that accuracy of tracking unpredictable signals, which relies heavily on feedback control, was similar to healthy adults. Clark and Robin (1998) also raised the likelihood of a “resource demand” explanation for visuomotor tracking deficits in AOS. Specifically, they demonstrated a trade-off between accuracy of absolute movement amplitude/duration (i.e., motor programming) and relative amplitude/duration (i.e., parameterization), that suggested a more limited working buffer that is less able to handle both processes (see also Klapp, 1995, 2003; Maas et al., 2008).

While these findings on their own may not establish a common cause for both the speech and visuomotor tracking deficits noted in the AOS participants, we have hypothesized that the two deficits are related and reflect impairment to similar orofacial motor control mechanisms (e.g., Ballard et al., 2003; Graziano and Aflalo, 2007). For example, it is argued that the premotor cortex underpins programming of sensory-guided movements generally (e.g., Roland et al., 1980a,b; Graziano and Aflalo, 2007). Consistent with this, Wang and Robin (1997) and Maas et al. (2008) found similar deficits across limb and speech motor programming tasks within individuals with AOS. Further, Robin et al. (2008a) showed that visuomotor tracking performance is significantly correlated with listener ratings of speech intelligibility and articulatory precision.

### Sensorimotor perturbation

The handful of sensorimotor perturbation studies of AOS appear to support the second explanation of the visuomotor tracking experiments offered above: sensorimotor integration is disrupted in AOS. Perturbation paradigms are used in many fields to understand how systems function. As a task is learned or performed, a perturbation is introduced and the response of the system is measured. By varying the timing, direction, frequency, and magnitude of sensorimotor perturbations during speech, we can study the integrity of the feedforward and feedback components of the speech motor control system. Perturbations used to study normal and disordered speech motor control have included bite blocks to prevent jaw movement (e.g., Ballard et al., 2004; Jacks, 2008; Golfinopoulos et al., 2011), mechanical shift in jaw trajectory during speech (Tremblay et al., 2003); intermittent downward pressure on the lower lip to prevent closure on bilabial plosives (Gracco and Abbs, 1985; de Miranda Marzullo et al., 2010), auditory masking to block feedback during speech production (Villacorta et al., 2007; Maas et al., 2013; Jacks and Haley, 2014; Maas et al., 2014), pitch or formant shifts to alter perceived quality or identity of vowels (e.g., Villacorta et al., 2007; Flagmeier et al., 2014; Terband et al., 2014), and temporal perturbations to alter perceived duration of segments (Cai et al., 2014).

Specifically investigating the integrity of feedforward movement commands generated by apraxic speakers, Maas et al. (2014) masked auditory feedback during speech, forcing them to rely on feedforward control. Maas et al. proposed that if apraxic speech errors are due to corrupted feedforward commands, masking auditory feedback would reduce the distance or contrast between fricatives (i.e., spectral mean ratio for initial “s” and “sh” in words). However, if the errors are due to the disruptive influence of auditory feedback, the masked condition would result in greater fricative contrast. Findings supported the latter feedback hypothesis, with increased contrast in the masked vs. unmasked condition for individuals with AOS relative to controls. These findings appear to support the hypothesis that sensory feedback interferes with speech production in persons with AOS. This is consistent with findings from visuomotor tracking studies described above that demonstrated interference of ongoing predictable movement patterns when persons with AOS had access to feedback. However, similar to Ballard and Robin (2007), the authors acknowledged that the higher response error rate in the AOS participants was also suggestive of an abnormal underlying program of movement (see also Maas et al., 2013; Jacks and Haley, 2014).

### Reaction time

Further support for the explanation of AOS as an impairment at some level of motor programing comes from our reaction time studies (Maas et al., 2008) driven by Klapp’s model of motor programming (Klapp, 1995, 2003). This model addresses both the specification of individual programs of movement and the process of sequencing and fluently transitioning between multiple motor programs. Klapp proposed that, when planning movement, the motor system first builds the internal structure (INT) of a motor program (e.g., a syllable), by selecting a stored program and modifying it for the given context; it then generates the required sequence of motor programs (SEQ) for a multi-unit production such as a polysyllabic word or a phrase. Applying reaction time methods, studies have found support for AOS as an impairment of INT processes; that is, in developing and retrieving individual motor programs rather than in the processes of sequencing multiple units (Deger and Ziegler, 2002; Maas et al., 2008). Briefly, the participants of Maas et al. (2008) produced a single syllable “ba” or a sequence of four “ba” syllables varying in temporal structure (short-long-long-short or long-short-short-long, where short was 150 ms and long was 450 ms). A “self-select” reaction time paradigm was applied; (a) the participant is cued to produce one of the four responses and when they are ready they press the spacebar on the keyboard: the lag from presentation to key press is called study time and measures time for planning in the INT stage; (b) the software then inserts a variable lag between the key press and a “go” signal and then time from the go signal to the onset of vocalization for the response is called reaction time and, in the four-syllable conditions, measures compiling of the sequence prior to execution (SEQ). For AOS participants, study time was protracted relative to control participants but reaction time was not, indicating impairment in selecting and preparing the syllable-sized motor program. While this study is not examining feedforward control through rapid integration of feedback during an utterance, as done in perturbation studies, the findings are consistent with “damage” to the motor programs or access to them in AOS (see Ames, 2009 below).

In summary, a variety of experimental paradigms have been used to investigate the nature of the speech motor control impairment in AOS. Currently, the evidence suggests that both feedback and feedforward control systems may be affected but additional work is required to examine which aspects of these systems are affected or whether some behaviors reflect altered functioning of intact network components that are receiving aberrant input from a damaged component. Questions such as these can be addressed with complementary neuroimaging and computational modeling studies to investigate the neural mechanisms that underlie AOS. Below, we review the neuroimaging and computational modeling studies of AOS to date and discuss their potential for improving our understanding of this disorder.

## Neuroimaging and anatomical substrates of AOS

Historically, AOS has been associated with lesions to BA44/45. This likely reflects the predominant etiology of stroke and the common concomitance of Broca’s aphasia. In 1996, attention was diverted away from BA to the anterior insula. Dronkers (1996) reported a lesion overlap study arguing that all patients in their sample who were diagnosed with AOS had damage to the anterior insula. While more recent studies have frequently reported insula involvement with these patients, several well-executed studies and reviews have cast doubt on a central or specific role in speech praxis (see Ziegler, 2008, for a review). Studies using perfusion-weighted imaging (Hillis et al., 2004), structural MRI analysis (Richardson et al., 2012; Whitwell et al., 2013; Ballard et al., 2014), and functional imaging with FDG-PET (Whitwell et al., 2013) have shown that damage or hypoperfusion in the inferior frontal gyrus (IFG) and lateral premotor cortex (BA44/BA6), rather than anterior insula, strongly predicts presence and severity of AOS. Ventral premotor cortex has been identified as central to articulatory preparation processes (Ackermann and Ziegler, 2010; Cai et al., 2014) and in generating the articulatory planning impairment in AOS (e.g., Robin et al., 2008b; Josephs et al., 2012, 2013; Whitwell et al., 2013; Ballard et al., 2014).

Some of the confusion and uncertainty in lesion mapping studies has come from this common co-occurrence of aphasia and the challenge of separating signs and lesions specific to AOS. A handful of studies have been able to examine lesion sites in relatively isolated cases of stroke-related AOS. In a review of three studies including a total of seven patients with pure AOS (Robin et al., 2008b), the only region of overlap in six of seven participants was ventral premotor cortex (BA6). Robin et al. proposed that left premotor cortex plays a critical role in speech motor programming and in generating the behavioral profile of AOS. Such lesion-symptom mapping approaches are useful for guiding hypotheses in computational modeling approaches exploring normal speech motor programming and the impact of AOS-associated lesions on network function; examples of this approach using the DIVA model are discussed below.

More recently, a progressive form of AOS (Josephs et al., 2012) has received considerable attention because it occurs in isolation (i.e., without concomitant aphasia) far more frequently than stroke-related AOS. Also, because the disease causes gradual atrophy of tissue, the functioning of the affected areas can be examined at different stages of progression. Individuals presenting with progressive AOS plus aphasia have been shown to have atrophy and hypometabolism in left frontal and temporal lobes, particularly the inferior, middle and superior frontal gyri and left insula (Nestor et al., 2003; Gorno-Tempini et al., 2004; Josephs et al., 2006; Rabinovici et al., 2008). Individuals with isolated progressive AOS, on the other hand, appear to have atrophy centered on the supplementary motor area and lateral premotor cortex (Josephs et al., 2012). Using region of interest analysis (10 mm radius) centered on the superior lateral premotor cortex, Whitwell et al. (2013) reported that gray matter volume in left lateral premotor cortex of individuals with isolated AOS correlated significantly with an AOS severity rating measure. Ballard et al. (2014) provided further support by reporting that gray matter volume in these areas was correlated with an index of temporal control of speech that is impaired in AOS but not aphasia. This measure, the pairwise variability index, is a normalized measure of relative vowel duration in adjacent syllables within multisyllabic words and serves to quantify the perception of equal stress commonly noted in apraxic speech.

In summary, consensus is building that the critical area damaged in both the stroke and progressive forms of AOS is along the lateral precentral sulcus, encompassing the posterior IFG (BA44) and lateral/ventral premotor cortex (BA6). Behavioral studies of AOS suggest this damage impairs feedforward motor control. There remain open questions regarding the nature of this disruption. Most current theories of motor control posit a tight coupling of feedfoward and feedback control systems: the development and maintenance of motor programs requires monitoring and integrating sensory feedback. Thus a stroke could impair the speech motor programs themselves or it could prevent the normal interaction of sensorimotor systems. The localization studies described above, while fundamental for understanding the disorder, cannot distinguish these possibilities. Rather, an increased focus on the *functional* contributions of the affected regions to the speech motor control network, and how interactions within this network are impacted by the damage, is an essential component to understanding normal and impaired speech production, including AOS. Improved neuroimaging methods and analyses now allow us to see how both functional and structural connectivity within the speech network differs in persons with disordered speech. By coupling these methods with a systems-level computational model of the sensorimotor interactions that underlie speech motor control, we can begin to construct a mechanistic understanding of speech disorders like AOS. Below, we review efforts in both of these areas specific to informing theories of AOS.

## Computational modeling approaches to AOS

Computational modeling can be used to explore the role played by different regions in the speech network as well as their interactions and impact on the speech network more broadly. In doing so, it can provide a bridge from studies of brain-behavior and lesion mapping to studies of functional and structural connectivity within this network. The DIVA model (Guenther, 2006; Guenther et al., 2006; Tourville and Guenther, 2011) provides a unified quantitative account of a wide range of speech production phenomena and neuroimaging data. It consists of feedforward and feedback control systems that are tuned by integrating motor output and sensory feedback, providing a comprehensive framework for interpreting studies of feedback and feedforward control in AOS. Each functional unit, or module, in the model has a hypothesized link to a specific neuroanatomical substrate based on human clinical, neuroimaging, and micro-stimulation studies and on non-human primate neural recording tracing studies (see Guenther et al., 2006; Tourville and Guenther, 2011). Simulations of a given speech task generate acoustic (e.g., formant frequencies), somatosensory (e.g., muscle positions), and brain activity predictions that can then be compared to empirical data in a straightforward manner. As such, it has been applied in numerous studies of disordered speech production (e.g., Max et al., 2004; Bohland and Guenther, 2006; Perkell et al., 2007; Ghosh et al., 2008; Tourville et al., 2008; Terband et al., 2009, 2014; Golfinopoulos et al., 2011; Cai et al., 2012, 2014; Civier et al., 2013). Such studies, in turn, are used to further refine the dynamics of the model and the mapping of model components to brain substrates.

Central to the DIVA model is the *speech sound map*, which is hypothesized to lie in left lateral premotor and adjacent posterior inferior frontal cortex (BA6 and BA44), the region most consistently associated with AOS lesions. Of note, cells within the speech sound map represent speech motor programs, or predictive forward models, for highly practiced speech sounds. They can represent any frequently produced speech sound, including single phonemes, syllables, or common multisyllabic words or phrases (e.g., “banana” or “I don’t know”) because with practice, smaller units are concatenated into larger ones. In the model, production of one of these well-learned speech units begins with the activation of the corresponding cell in the speech sound map. Signals sent from the activated speech sound map to primary motor cortex (directly and through the cerebellum), represent feedforward commands that encode the time series of articulator movements that produce the desired speech sound. The motor programs in the speech sound map are therefore similar to those described by others (e.g., generalized motor programs of Schmidt and Lee (1999); gestural scores of Browman and Goldstein (1989)). Through additional downstream processes, parameters such as movement rate and absolute force can be modified based on the demands of the speaking context.

Cells of the speech sound map also project to auditory and somatosensory cortices. These projections encode the sensory target, or forward models, associated with the speech motor program represented by that cell, i.e., the desired speech sound. During speech production, afferent auditory and somatosensory feedback is continuously compared to this target; any discrepancy between expected and actual sensory information constitutes an error that is transformed into corrective movements via projections from the sensory areas to the primary motor cortex. The corrective movement is also used to update the feedforward commands that generated the erroneous movements, i.e., the signals from the speech sound map to motor cortex. Early in development the feedforward command is inaccurate, and feedback is used to improve movement accuracy over repeated attempts (e.g., Kawato, 1999). With repeated practice, there is a shift to reliance on highly accurate and rapid feedforward control.

The speech sound map thus represents the interface between the speech feedback and feedforward control systems, linking sensory goals to the motor commands that can achieve them (analogous to what Rizzolati and many others have described as “mirror neurons”, e.g., see Rizzolatti and Craighero, 2004). The model is therefore particularly well-suited for exploring the neural mechanisms that underlie AOS, providing a unified framework for generating and testing hypotheses and synthesizing existing knowledge of behavioral, neuro-anatomical and neuro-functional changes. By mapping speech features and lesion sites in AOS onto model components in the DIVA model, we can (a) develop a model-based explanation of the impairment; and (b) design computational modeling studies that systematically alter the integrity of model components and/or connections between components. Through this process, we can validate model-based explanations but also undertake more controlled studies of speech praxis than are possible with heterogeneous patient samples.

Only two studies to date have reported DIVA computer simulations of apraxic speech (Ames, 2009; Terband et al., 2009). When Ames “damaged” the speech sound map, the synthesized speech output became nonfluent with phoneme distortions, schwa insertion, movement of syllable boundaries, vowel prolongations, and related prosodic alterations. That is, the model’s output simulated AOS speech features (McNeil et al., 2009) consistent with Aichert and Ziegler’s (2004) damaged motor program hypothesis of AOS. Damage to the inferior frontal sulcus component, on the other hand, did not appear to affect fluency of productions and generated few speech errors at the single word level, with these typically being phoneme sequencing errors. This study did not experimentally examine the impact of the damaged component on the functioning of the whole network or other intact network components. However, the resulting speech features were considered consistent with the lesion having its impact on the integrity of learned feedforward commands; specifically, motor programs may be degraded or corrupted, have higher threshold of activation, or be more resistant to modification in response to feedback. These findings were verified with a stroke case presenting with a lesion including the frontal operculum but leaving inferior frontal sulcus intact (Ames, 2009).

The second study to perform computational simulations of AOS considered the childhood form. Terband et al. (2009) used computer simulations to explore whether a shift in the balance of feedforward and feedback contributions to motor output could explain the speech characteristics of childhood AOS. The relative weighting on feedforward vs. feedback control was varied from 90:10 to 55:45 as the model learned novel vowel-consonant-vowel sequences (e.g., “uba”). The acoustic output of model simulations was evaluated and found to approximate childhood AOS of increasing severity as weightings shifted from 70:30 to 54:45, supporting the authors’ hypothesis of childhood AOS representing an imbalance away from the normal 90:10 weighting. They noted that impaired development of accurate feedforward commands could also be due to reduced sensitivity of oral structures or increased neural noise throughout the system, which would imply inaccurate error detection and correction mechanisms (i.e., impaired feedback processes) as well. Importantly, carefully designed behavioral perturbation paradigms developed by Guenther et al. and described above should enable us to tease apart these different control mechanisms and impairments in future studies. For instance, according to the model, increased influence of feedback-based commands on motor output simulated by Terband et al. would manifest itself as greater functional coupling of sensory error maps in superior temporal and lateral parietal cortex with lateral premotor cortex, particularly in the right hemisphere (see Tourville and Guenther, 2011), even in the absence of sensory error and concomitant activity in those regions. In other words, the apraxia symptoms simulated by Terband et al. (2009) may result from atypical interactions within the speech motor control network that are not necessarily accompanied by changes in local activity in the network. By applying network connectivity methods, in complementary studies we can test this type of model-derived hypothesis. In the following section, we discuss how these methods may be applied to the study of AOS.

## Network connectivity in AOS

A number of methods have now been developed for analyzing structural, functional, and effective network connectivity and have been used for understanding normal and disordered speech production. These methods allow us to move beyond questions of where the damage is to questions of how the damage is impacting the speech network. Structural connectivity methods provide a means of estimating the actual connectivity between regions within a functional network. Diffusion tensor imaging (DTI) is now a relatively common method that measures the diffusion of water molecules within tissues, in particular white matter fibers in the nervous system. With measures of magnitude, anisotropy, and orientation of diffusion, fiber-tracking methods reveal the integrity of the white matter connections in a functional network. Graph theory can then be applied to model pairwise relations between connected nodes in these white matter networks; defining the degree of connectivity, the nodes that are serving as structural hubs in the network, and the efficiency of information flow in the network based on path lengths between nodes (Rubinov and Sporns, 2010; Cai et al., 2014). Functional connectivity methods, on the other hand, provide a means of assessing how tightly coupled regional *activity* is within a network. Resting state or task-based fMRI data are analyzed for *functional* connectivity using correlations and provide insight into which regions are firing together and therefore coupled. Effective connectivity analysis methods (i.e., structural equation modeling, SEM, or dynamic causal modeling, DCM) then estimate the strength and direction of influence between activated regions in a network and provide a powerful method for detecting which areas or connections are driving functional changes.

Studies have begun to explore how interactions within the speech motor control network change when the normal balance of feedforward and feedback-based control during speech is manipulated. For instance, using structural equation modeling, Tourville et al. (2008) demonstrated the dynamic nature of the healthy speech motor network in response to relatively brief perturbation. They showed that strength of connectivity between bilateral auditory cortical areas and right frontal areas varied in response to perturbing auditory feedback of the speaker’s own voice; that triggers a compensatory response in vocal tract configuration to oppose the perturbation. Such data show the strength of a connectivity approach for examining both brief perturbations and the more permanent perturbations arising from neurological damage. Further elucidating the role of the right-hemisphere network components, Golfinopoulos et al. (2011) used effective connectivity analyses to show that feedback-based correction of articulatory motor commands after somatosensory perturbation integrally involves right ventral premotor and inferior frontal cortex as well as the traditionally associated left IFG. While not focused specifically on AOS, studies such as these lay the groundwork for the types of changes in the speech motor control network we might expect in persons with AOS, if the disorder is associated with an overreliance on feedback-based control.

At the time of writing, we are unaware of any studies directly examining changes in functional connectivity with AOS. The disorder of stuttering, however, has received some attention and studies are demonstrating both functional and structural differences in the speech motor control network relative to non-stuttering speakers (e.g, Sommer et al., 2002; Watkins et al., 2007; Lu et al., 2009, 2010; Cykowski et al., 2010; Chang et al., 2011; Howell et al., 2012; Xuan et al., 2012; Cai et al., 2014). While stuttering is distinct from AOS, theoretical explanations of the disorder have centered on altered reliance on feedback vs. feedforward motor control, similar to AOS. On one example, Chang et al. (2011) reported reduced structural and functional connectivity between left BA44 and premotor areas, in adults who stutter relative to control speakers, but *increased* connectivity between right-hemisphere homologues. Functional connectivity between BA44 and auditory cortex was normal, suggesting a feedforward, rather than feedback, control deficit in stuttering. Further exploration of the network connectivity differences between various speech disorders such as stuttering and AOS will improve the specificity of existing models of speech motor control.

Another approach to investigating connectivity that complements the DIVA computational modeling approach and direct connectivity studies in patients is represented in the study of Eickhoff et al. (2009). Eickhoff et al. used a combination of meta-analyses to identify brain regions that were active during speech production across a wide range of tasks and studies. Using dynamic causal modeling of fMRI imaging data collected during a verbal fluency task, they tested various connectivity configurations of these areas. The configuration with strongest support from their analyses had BA44 activating the insula, which then activates BA6 via parallel connections through the cerebellum and caudate. The authors proposed that BA44 is activated after language-based word retrieval processes, presumably once the phonological representation of a syllable or word is available. The insula is then activated with the role of generating the phonetic plan for coordinating multiple articulators. Parallel connections from the insula to the cerebellum and basal ganglia appear to represent preparatory processes, consistent with the traditional view of these latter subcortical structures in speech motor programming loops (Duffy, 2005) and in feedforward motor control (Guenther et al., 2006). Output from these pathways is fed forward to the premotor cortex (BA6) and primary motor cortex for final execution. In this model, the premotor cortex has a role in converting movement patterns into muscle-specific commands.

Consistent with the meta-analytic approach, Eickhoff et al.’s (2009) finding is heavily dependent on the regions of interest and analyses reported in the original studies and the connectivity models that were tested. Models that included a direct pathway from BA44 to the premotor cortex or a starting point other than BA44 were not considered, and some areas known to be involved in the speech motor network were not included. Nevertheless, the resulting model identifies a specific role in the network for anterior insula and a clear statement about the coupling properties between the insula, BA44, and BA6 that can be directly tested in AOS with competing theories and complementary approaches of behavioral, computational modeling, and neural connectivity studies.

## Conclusions

The use of computational modeling and network connectivity analyses is relatively rare in the field of AOS. By coupling these two approaches along with behavioral perturbation studies we will have the strongest evidence to construct a mechanistic account of AOS. Studies from healthy speakers and other speech disorders are demonstrating the valuable information that can be gleaned for understanding network dynamics in intact and impaired systems and how coupling properties may be differentially affected with damage to different network components. Such studies will further elucidate relationships between the different pathways within this complex network. An understanding of the connectivity and interactions will move us toward understanding the apparent heterogeneity in this patient population, potential subtypes related to factors such as disease process, and critical differences against other motor speech disorders.

With an understanding of the components of the speech motor network that are damaged vs. spared in AOS, both structurally and functionally, we will gain insight into the relationships between the changes and the resulting symptomatology. With this information, intervention researchers will be better positioned to recognize prognostic indicators and develop treatment approaches that maximize the performance of the altered network through targeted and intensive behavioral exercises that can modify connectivity (Kleim and Jones, 2008). In addition, we will better understand the mechanisms by which emerging cortical stimulation methods (e.g., Meinzer et al., 2012) can excite or inhibit network components to facilitate functioning within the broader network and amplify the effects of behavioral exercises.

## Conflict of interest statement

The authors declare that the research was conducted in the absence of any commercial or financial relationships that could be construed as a potential conflict of interest.
